# The Characterization of the Morphological and Molecular Traits of *Phaseolus coccineus* in the Aniene Valley: Insights into Genetic Diversity and Adaptation

**DOI:** 10.3390/plants13233320

**Published:** 2024-11-26

**Authors:** Mario Ciaffi, Anna Rita Paolacci, Martina Marcomeni, Lorenzo Coluccia, Paola Taviani, Enrica Alicandri

**Affiliations:** 1 Department for the Innovation in Biological, Agrofood and Forestal Systems, Tuscia University, 01100 Viterbo, Italy; ciaffi@unitus.it (M.C.); arpaolacci@unitus.it (A.R.P.); martina.marcomeni@unitus.it (M.M.); lorenzo.coluccia@unitus.it (L.C.); 2 ARSIAL, Regional Agency for the Development and the Innovation of Agriculture in Lazio, Via Rodolfo Lanciani 38, 00162 Roma, Italy; paola.taviani.arsial@gmail.com

**Keywords:** *Phaseolus coccineus*, bean landraces, genetic diversity, SSR molecular markers, morphological traits, germplasm conservation

## Abstract

This study aimed to characterize the genetic diversity and morphological traits of 32 populations of *Phaseolus coccineus* collected from the Aniene Valley. Quantitative seed trait analyses revealed that Mandolone accessions exhibited significantly higher seed weights and dimensions compared to Fagiolone accessions. Specifically, Mandolone accessions showed an average weight of 189.48 g per 100 seeds, with seed heights and lengths averaging 14.38 mm and 23.84 mm, respectively. In contrast, Fagiolone accessions had an average seed weight of 174.13 g, with seed heights and lengths of 13.95 mm and 22.58 mm, respectively. Molecular analysis, conducted using 9 polymorphic SSR loci, identified 53 alleles across 320 genotypes, with a mean expected heterozygosity (He) of 0.695. This indicated that there was high genetic variability within the Aniene Valley populations. The genetic diversity analysis revealed two genetic clusters corresponding to the Mandolone and Fagiolone landraces. This was supported by UPGMA, PCoA, and STRUCTURE analyses. This study highlights the need to conserve the genetic diversity within these landraces and provides a basis for the development of conservation strategies for *P. coccineus* germplasms in the singular ecological context of the Aniene Valley.

## 1. Introduction

Among leguminous crops, *Phaseolus coccineus* L., commonly known as the scarlet runner bean, is recognized as the third most important bean species worldwide, following *P. vulgaris* (common bean) and *P. lunatus* (lima bean) [1,2,3]. *P. coccineus* is distinguished from its counterparts by its predominantly allogamous and perennial nature [4,5]. Recent genetic studies, including analyses by Angioi et al. [6], have identified distinct genetic groupings for wild runner beans, suggesting that there have been multiple domestication events in Mesoamerica. This region is acknowledged as the origin and center of domestication of this species, with both wild and cultivated forms coexisting [1,7]. Spanish and Portuguese sailors and traders introduced the runner bean to Europe after the colonization of the Americas, likely along with the common bean. It subsequently spread from the Iberian Peninsula to Italy and other parts of Europe [1,8].

This climbing perennial crop is frequently cultivated as an annual variety due to its green pods or dry seeds [7], which are known for their protein, dietary fiber, vitamin, and mineral contents [4,9]. Due to its distinctive colorful inflorescences, it is also grown as an ornamental climber plant [7].

In 2022, global dry bean production exceeded 28 million metric tons [10]. In Europe, major cultivation areas include the Iberian Peninsula, France, Italy, Greece, and the Balkan regions. These areas collectively produced approximately 540,000 tons in 2017 [10].

In 2023, Italy contributed to bean production with a yield of 10,480 tons of dry beans, grown over an area of approximately 5000 hectares. 

The Italian regions most involved in bean cultivation are Campania, Piemonte, Veneto, Emilia-Romagna, and Lazio [11]. In Lazio, farmers have preserved and cultivated a wide variety of bean landraces over generations, selectively adapting them to distinct environmental conditions [12]. The traditional landraces, known for their natural genetic diversity, are often incompatible with commercial agricultural practices, leading to a significant decline in output over the past century [13]. The preference for uniform, high-yielding commercial varieties has often seen the replacement of traditional landraces, leading to reduced genetic variability [14,15]. Genetic erosion diminishes crops’ ability to adapt to environmental changes, pests, and diseases, posing a threat to food security and cultural heritage [14]. To mitigate these effects, both in situ (on-farm) and ex situ (gene bank) conservation strategies can be adopted, along with the provision of support for farmers who maintain traditional practices and cultivate diverse crop varieties [15].

In alignment with the objectives of the Convention on Biological Diversity (UNCBD, Rio de Janeiro, 1992), the Lazio Region of Italy enacted Regional Act No. 15 on 1 March 2000, entitled “Protection of Autochthonous Genetic Resources of Agricultural Interest”. This legislation, enacted to protect and promote the autochthonous genetic resources of the region, established ARSIAL (Regional Agency for the Development and Innovation of Lazio Agriculture) to implement protection measures. ARSIAL manages two functioning instruments: the Regional Voluntary Register (RVR), which catalogs indigenous genetic resources of agricultural interest (covering both plant and animal resources) based on evaluations by scientific commissions, and the Conservation and Safety Network (CSN), which supports the in situ and on-farm conservation of registered genetic resources, promotes their reintroduction into cultivation, and assists farmers in promoting their propagation and sustainable use [16].

The Aniene Valley, located in the eastern–central part of the Lazio Region in Italy, is a significant site in terms of the cultivation of various crops, including several distinct bean landraces. Its peculiar agroecological conditions, such as its diverse microclimates, the rich soil composition, and the orographic conditions, as well as the type of agriculture, featuring practices based on low-impact agronomic techniques, provide an optimal environment for the growth and adaptation of these species [17]. The Aniene Valley, covering approximately 600 km^2^, displays diverse forests and fauna, and exhibits agrarian biodiversity, characterized by three distinctive ecological and landscape elements: the Simbruini Mountains Regional Park, the largest protected area in Lazio; the Lucretili Mountains Park; and the entire Aniene River basin, a major tributary of the Tiber River [13]. The Upper Aniene Valley hosts a unique array of plant species, including several endemic species, and is shaped by adaptation to the valley’s distinct geology and microclimate. This region combines productive agricultural lands with diverse ecosystems—riparian zones along the Aniene River, deciduous and mixed woodlands, and high-altitude grasslands—that contribute to soil health, water retention, and resilience of local agricultural systems. The frequent rainfall further supports the Aniene Valley’s role as a critical water basin. Additionally, the Mid and Upper Aniene Valley, particularly near the Simbruini Mountains, contributes to climate regulation through extensive forest cover that sequesters atmospheric CO_2_. Recent forest management practices, including shifts from coppicing to high-forest systems, have increased biomass storage and soil carbon levels, further enhancing the valley’s role in sustainable land management and agricultural productivity [18].

Significantly, within this area, the cultivation and consumption of beans, integral to local communities, is deeply ingrained in traditional customs and cultural practices [19]. Bean landraces are not only shaped by natural selection within their growth environment, but also by traditional mass selection methods practiced by farmers [20]. This ancient form of genetic improvement involves the selection of individuals within a population that display advantageous phenotypes, whose seeds are then combined to create a new population with rich genetic variability. This approach contrasts sharply with modern cultivation practices, which often result in a narrower genetic base due to intensified artificial selection [20]. 

The RVR includes a single *Phaseolus coccineus* landrace, cultivated in the middle part of Aniene Valley area, that is commonly known as “Fagiolone di Vallepietra” (https://www.arsial.it/biodiversita/registro-volontario-regionale/ last accessed on 23 October 2024).

In this study, we aim to comprehensively characterize the morphological and molecular diversity of the *Phaseolus coccineus* populations cultivated in the Aniene Valley. By integrating morphological traits analyses and molecular markers studies, we seek to elucidate the population structure of *Phaseolus coccineus* within this singular ecological context. Our findings hold implications for both agricultural practices and biodiversity conservation, supporting the sustainable management and utilization of *P. coccineus* genetic resources in the Aniene Valley and similar environments.

## 2. Results and Discussion

### 2.1. Seed Morphological Analysis

With regard to the four quantitative traits related to seed (the weight of 100 seeds, length, height, and their ratio), the Mandolone accessions exhibited higher seed weight and dimensions compared to those of the Fagiolone accessions (Table S1 and Figure S1).

For Mandolone seeds, the weight of 100 seeds varied between 180 g for Fill 5 and 201 g for accession Fill 12, with an average of 189.48 g across the 16 accessions. This was significantly higher than the weight observed for the 16 accessions of Fagiolone (174.13 g). For Fagiolone seeds, the weight of 100 seeds varied between 160 g for accession Vall 11 and 188 g for accession Vall 14 (Table S1 and Figure S1). Similarly, the average height and length values of the seeds in the 16 Mandolone accessions (14.38 and 23.84 mm, respectively) were significantly higher than those recorded for the same quantitative seed parameters in the 16 accessions of Fagiolone (13.95 and 22.58 mm, respectively) (Table S1). For Mandolone, the variation in height and length ranged between 13.55 mm and 15.20 mm and between 22.55 mm and 25.20 mm, respectively, with both minimum and maximum values recorded in the Fill 6 and Fill 12 accessions. In Fagiolone, the minimum height and length values (13 mm and 21.50 mm, respectively) were observed for accession Subi 1, while the maximum values (15 mm and 23.80 mm, respectively) were found for accession Vall 14 (Table S1 and Figure S1). Finally, the mean value of the L/H ratio recorded for the accessions of Mandolone was slightly higher (1.66) than that determined for the Fagiolone accessions (1.62), although the differences between the means were not statistically significant (Table S1). Seed parameters, including the weight of 100 seeds, height, and length, were selected for their agronomic significance as these characteristics directly influence yield potential and resilience in the local cultivation environment [13]. The results clearly indicated that seed morphology is a valuable marker in terms of distinguishing different local varieties, as shown in previous studies [21,22]. 

### 2.2. Genetic Analysis Using SSR Molecular Markers

Among the 12 SSR loci selected in order to analyze variability within and among the populations of *P. coccineus* collected in the Aniene Valley, 2 loci (AZ301573 and X96999) were found to be monomorphic, and 1 locus (AZ301561) exhibited an amplification profile that was difficult to interpret. Therefore, Table S2 presents the main genetic parameters that were calculated for the remaining nine polymorphic loci. In the 320 analyzed genotypes (10 individuals from 32 populations), a total of 53 alleles were identified, with an average number of alleles per locus of 5.89, ranging from 2 for locus U18349 to 10 for locus X80051 (Table S2). Aside from the latter, the loci with the highest number of alleles were M75856, X74919 (7), and X04001, X60000, and AF483902 (5 each). Four of the six loci displaying the highest number (X80051, M75856, X60000, and AF483902) were also the most polymorphic, as indicated by the low frequency of the most represented allele (Table S2). The expected heterozygosity (He) values ranged from 0.516 (U18349) to 0.876 (X80051), with five loci (X80051, M75856, X79722, X60000, and AF483902) exhibiting values higher than the mean of 0.695 (Table S2). 

These results highlighted that *P. coccineus* accessions from the Aniene Valley, despite their limited cultivation area, exhibit a high level of genetic variability. This is particularly evident when comparing the genetic diversity parameters obtained for this *P. coccineus* collection. These were determined through the analysis of 12 SSR loci in a broad collection of 148 European *P. coccineus* accessions [2]. The average number of alleles per locus (Na) obtained for the Aniene Valley collection was slightly lower than that determined for the 148 European accessions (Na = 5.89 vs. 8.3) [2]. However, the average expected (He) and observed (Ho) heterozygosity values were significantly higher for the Aniene Valley accessions (He = 0.695; Ho = 0.406) than for 148 European accessions (He = 0.370; Ho = 0.12) [2].

The discriminatory power of each of the nine SSR markers, measured through PIC values, was relatively high, with an average value of 0.663. Eight out of nine loci exhibited PIC values > 0.5 (Table S2). The most informative loci were X80051, M75856, and X79722, which demonstrated the highest PIC values (0.867, 0.815, and 0.758, respectively) and expected heterozygosity (He) values (0.876, 0.836, and 0.788, respectively), as well as the lowest MAF values (0.272, 0.231, and 0.294, respectively) (Table S2). The main genetic parameters determined for the 32 populations are presented in Table 1, with the results revealing that the number of alleles per locus (Na) ranged from 2 for the Trev_2 population to 3.222 for the Fill_2 population. The number of effective alleles varied from 1.577 for the Vall_13 population to 2.581 for the Fill_2 population. Out of the 53 alleles identified in the 32 populations, 8 were private alleles. Two of these were found in the Fill_2 population and one was discovered in each of the following populations: Vall_2, Vall_9, Vall_11, Vall_12, Vall_14, and Fill_11. The rare and private alleles discovered in *P. coccineus* accessions are reported in Table S3. 

The expected heterozygosity (He) values ranged from 0.274 for the Vall_13 population to 0.593 for the Fill_2 population, with 16 populations (Fill_2, Fill_10, Fill_3, Fill_4, Fill_1, Fill_5, Fill_12, Subi_1, Fill_13, Fill_11, Vall_6, Subi_2, Fill_7, Vall_1, Vall_5, and Vall_2) exhibiting values exceeding the mean He value of the 32 populations (0.441). Consistent with He values, the same 16 populations also showed the highest Shannon Index (I) values (Table 1). Our He values were higher than those found by Bosmali et al. [23], in a collection of 14 *Phaseolus coccineus* landraces from Greece, using 14 microsatellite markers (SSR and EST-SSR). The expected heterozygosity (He) values ranged from 0.164 (Gigantes population from Prespes Lake area) to 0.407 (*P*. *coccineus* population from Arkadia). This suggests that there is a greater level of genetic diversity within the populations cultivated in the Aniene Valley, which is potentially due to differing environmental conditions or historical management practices that have contributed to the conservation of genetic variability.

The genetic diversity observed in *Phaseolus coccineus* accessions by assessing SSR markers is found in loci associated with genes involved in various adaptive and functional roles. For example, PV-ag004 (X04660, Tables S2 and S4), identified as a pseudogene for phytohemagglutinin, may be involved in defense mechanisms against herbivores or pathogens [24]. While phytohemagglutinins are traditionally associated with plant defense, their pseudogene counterparts may indicate the evolutionary adaptation of *P. coccineus* in specific environments [24]. Finally, loci associated with the Phaseolin G-box binding protein, such as U18349 (Tables S2 and S4), potentially have roles in regulating seed storage proteins. This may influence nutritional qualities and seed viability under heterogeneous environmental conditions [25].

To detect potential deviations from the Hardy–Weinberg equilibrium (HWE), occurring due to an excess or deficit of heterozygotes, the inbreeding coefficient (Fis) was calculated. Significant deviations from the HWE, attributed to a deficit of heterozygotes, were observed in 22 out of the 32 analyzed populations, with Fis values ranging from 0.123 for the Vall_10 population to 0.424 for the Fill_11 population (Table 1). Excluding the locus X04660, which exhibited a negative Fis value, all other loci contributed to these deviations, which likely occur due to an excess of homozygotes (Table S2). 

These results are somewhat unexpected, considering that *P. coccineus* is predominantly an outcrossing species, and populations of this species are generally expected to adhere to the genotypic proportions predicted by the HWE. However, similar studies on maize landraces, conducted using different molecular markers, have reported significant deviations from the HWE [26,27,28]. Experimental errors, null alleles, population substructuring, non-random mating, genetic drift, and farmer-driven selection may be involved in the homozygote excesses or deficits observed in outcrossing species [26,28]. While experimental errors cannot be entirely excluded, no evidence of null alleles was found in any of the nine loci analyzed. Therefore, inbreeding, genetic drift, and farmer-driven selection are considered potential factors that can explain the observed excess of homozygotes seen in this study. 

In particular, considering the small size of the sampled farms, which had from 100 to 500 cultivated plants, genetic drift could play a significant role in determining the deviations observed from the HWE. Moreover, under the specific cultivation conditions seen in the Aniene Valley, a higher level of self-fertilization may occur than is typically expected for an outcrossing species like *P. coccineus*. Finally, interviews with local farmers revealed that most of them select the best and largest seeds from the most vigorous plants in order to use them for sowing in the following year, thereby potentially altering the genetic structure of *P. coccineus* populations. This practice, repeated annually, may have increased the frequency with which alleles associated with the most important agronomic traits occur. Farmers may unknowingly select plants with a lower genetic load, which could subsequently exhibit a higher level of homozygosity than predicted using the HWE.

The components of genetic variability, determined through AMOVA, were analyzed for all 32 populations and for populations that were grouped based on their geographic origin (Vallepietra, Filettino, Trevi nel Lazio, and Subiaco) (Table 2). The AMOVA analysis indicated that most of the genetic variability detected by the nine SSR loci was found within populations (69%), with only 31% attributable to differences between populations (Table 2). The relatively low level of differentiation between populations is further supported by the low Fst index value (0.248) observed for the 32 populations (Table 2). Notably, the Fst value determined for the populations grouped by the four municipalities where Fagiolone is cultivated was lower than that determined for all 32 populations, and the same trend was observed for genetic variability between populations. This was determined by AMOVA (Table 2).

These findings may be explained by the exchange of seeds among farmers cultivating *P. coccineus* genotypes. Information gathered directly on site indicates that the practice of seed exchange appears to be relatively widespread among some farmers, especially those within the same municipality. This practice aims to increase the quantity of available seeds for the following year’s planting and, in some cases, to reintroduce *P. coccineus* into cultivation in farms where its use had been temporarily suspended.

**Figure 1:**
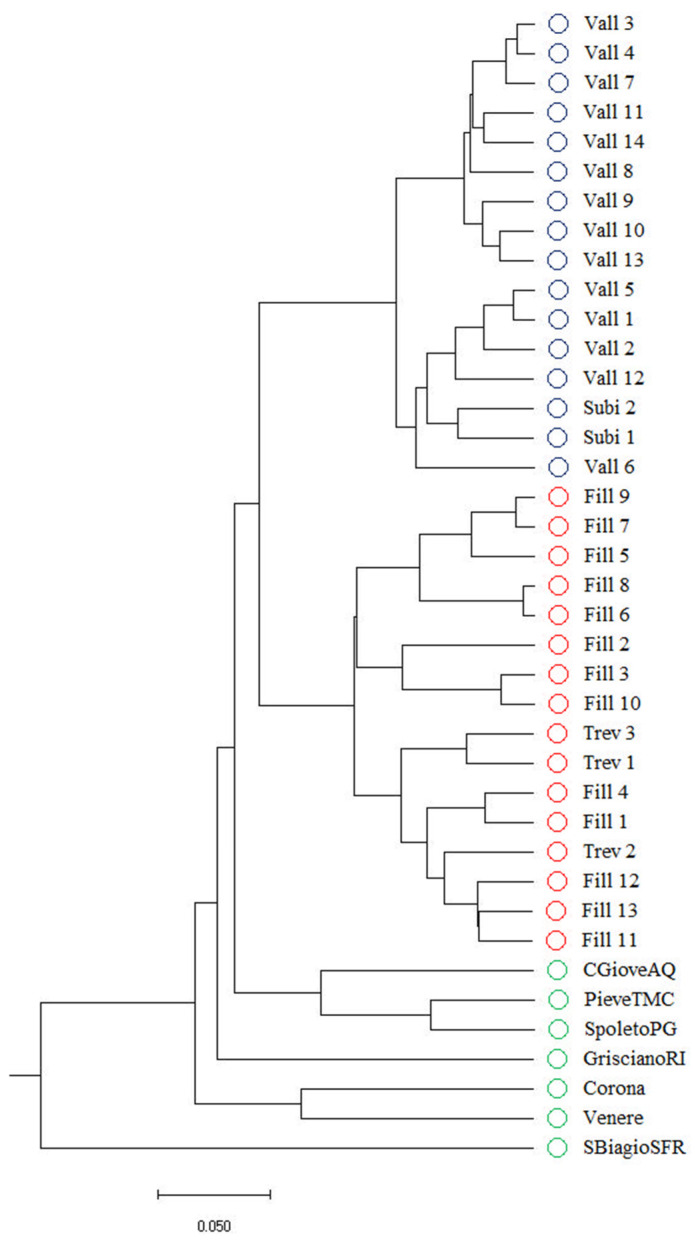
UPGMA dendrogram of the genetic relationships between the 32 accessions of *P. coccineus* collected in the Aniene Valley, labeled as Fagiolone (blue circles) and Mandolone (red circles), along with the 7 accessions used as controls (green circles).

The genetic relationships among the 32 populations of *P. coccineus* collected in the Aniene Valley and the 7 accessions of *P. coccineus* cultivated in Central Italy with a similar seed morphotype are illustrated in Figure 1. The dendrogram clearly shows that the accessions collected in the Aniene Valley are genetically distinct from the 7 control accessions, demonstrating their uniqueness and distinguishability from the *P. coccineus* germplasm cultivated in Central Italy. Moreover, the phylogenetic tree indicates that the 32 populations can be separated into two distinct clusters: the first comprising the 16 accessions, labeled with the name Fagiolone (blue circles), and the second including the remaining 16 accessions, labeled with the name Mandolone (red circles).

**Figure 2:**
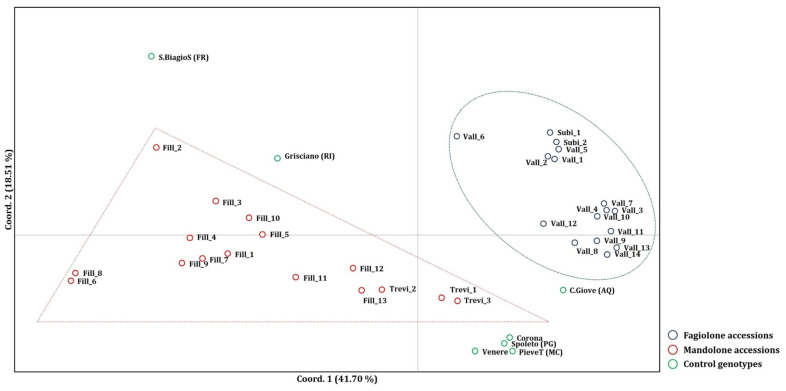
Principal component analysis based on genetic distances, determined using Nei’s. This was performed for the 16 populations of the Fagiolone landrace (blue circle) and the 16 populations of the Mandolone landrace (red circle), along with the 7 genotypes used in the analysis as controls (green circle). Coord 1: Principal Component 1; Coord 2: Principal Component 2.

These findings are further supported by Principal Coordinates Analysis (PCoA) and the determination of pairwise Fst values for each population collected in the Aniene Valley (Figure 2 and Table S5). Specifically, the PCoA indicates that the first two components represent 60.21% of the total variation, with the first component explaining 41.70% and the second 18.51%. The PCoA clearly demonstrates a well-defined cluster for the Fagiolone accessions and a more dispersed distribution for the Mandolone accessions. Both clusters are distinct from the seven control genotypes, which are scattered throughout the plot (Figure 2).

### 2.3. Genetic Structure of P. coccineus Accessions Collected in the Aniene Valley

**Figure 3:**
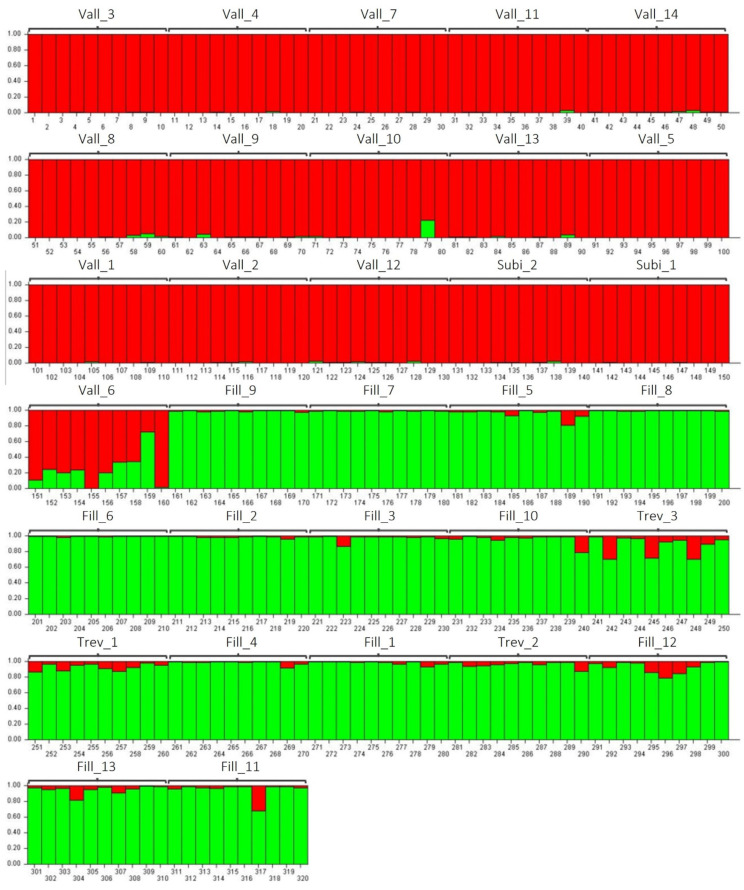
Bar plot depicting the average membership proportions (Q) of the 320 genotypes belonging to the 32 accessions of *P. coccineus* collected in the Aniene Valley for K = 2 (in red and green). Values were obtained through STRUCTURE analysis. Genotypes with a membership probability Q > 0.75 were assigned to the corresponding genetic groups (red or green).

The genetic structure of the *P. coccineus* germplasm in the Aniene Valley was analyzed using STRUCTURE v. 2.3.4 software. This allowed for the identification of the most probable number of genetic groups in the 32 populations, the assignment of each of the 320 genotypes to the identified genetic groups, and the identification of genotypes of mixed origin. The analysis indicated that the 320 genotypes can be divided into two genetic groups (K = 2) (Figure S2), which is consistent with the two main clusters obtained through UPGMA (Figure 1). The first genetic group (red) includes 157 of the 160 genotypes related to the 14 accessions collected in Vallepietra, as well as the 2 collected in Subiaco, belonging to the Fagiolone landraces (Figure 3). Three genotypes of the accession Vall_6 (numbers 157, 158, and 159 in Figure 3) can be considered genotypes of mixed origin. This likely results from hybridization events that occur between the two genetic groups, as they have membership probability values Q < 0.75 (Table S6). The second genetic group (green) includes 156 out of the 160 genotypes related to the 13 accessions collected in Filettino, and the 3 accessions collected in Trevi nel Lazio, belonging to the Mandolone landrace. Four genotypes, three from the Trev_3 accession (numbers 242, 245, and 248 in Figure 3) and one from the Fill_11 accession (number 317 in Figure 3), can be considered putative hybrids of the two genetic groups as they also have a Q < 0.75 (Table S6). The seven genotypes of mixed origin were excluded from subsequent analyses of genetic diversity between the two groups.

The STRUCTURE analysis clearly indicated that, in the *P. coccineus* germplasm cultivated in the Aniene Valley, two distinct landraces are present. These are consistent with the names assigned to different accessions by local farmers: Fagiolone and Mandolone. This result is somewhat surprising considering the similarities in seed morphotype between the Fagiolone and Mandolone accessions and the geographical proximity of the two municipalities where the two landraces are primarily cultivated, Vallepietra and Filettino, which are no more than 8 km apart. Considering that only two *P. coccineus* landraces from Lazio have been recognized as native genetic resources—Ciavattone di Grisciano and Fagiolone di Vallepietra—the clear genetic distinctiveness of Mandolone, as demonstrated in this study, may allow its registration in the RVR. 

The analysis of genetic diversity between the two groups corresponding to the two *P. coccineus* landraces in the Aniene Valley revealed that Mandolone exhibits higher values for the parameters Ne, I, Ho, and He than Fagiolone, although these differences were not statistically significance when assessed using the Kruskal–Wallis test (Table S7). Out of the 53 alleles identified in the 313 genotypes, 26 were specific to one of the two landraces, with both landraces studies showing the same number of private alleles (13) (Tables S7 and S8).

The AMOVA analysis indicated that 72% of the genetic diversity detected through SSR markers was attributable to differences within groups, while only 28% occurring due to differences between the two groups (Table S9).

The results of the genetic diversity analysis suggest that environmental factors in the Aniene Valley may drive genetic adaptation within these landraces. Specifically, the environmental conditions, such as altitude and soil composition, could create selective pressures that enhance genetic differentiation between Mandolone and Fagiolone. These pressures may lead to variation in adaptive traits, fostering resilience in response to local soil nutrient profiles, water availability, and microclimatic conditions. The distinct environmental conditions have likely facilitated the observed divergence, contributing to the adaptation of each landrace in the Aniene Valley.

## 3. Materials and Methods

### 3.1. Plant Material

The cultivation of *P. coccineus* is widespread in the Aniene Valley. A landrace named Fagiolone is grown in this area. This, along with the Fagiolo di Grisciano, is one of the two landraces of *P. coccineus* registered in the RVR. The seeds of this landrace have a large white confetti-like shape, hence the name Fagiolone (large bean) (Figure S3). Its cultivation is almost exclusively conducted on small plots in the municipality of Vallepietra in the province of Rome at an altitude of about 800 m above sea level. Its cultivation covers a total area of approximately one hectare. However, it is common to find the cultivation of this landrace in family gardens in many municipalities of the Aniene Valley, especially in Subiaco, Jenne, Arsoli, and Vallinfreda, all of which are located in Rome Province. In the two neighboring municipalities of Vallepietra, located in Filettino and Trevi nel Lazio, located within the province of Frosinone, the cultivation of a runner bean landrace, known as Mandolone, has been documented. Notably, this landrace exhibits seed traits closely akin to those of the Fagiolone landrace (Figure S3). The origins of Fagiolone and Mandolone are unknown, and despite the distinct names assigned by farmers to different accessions from various municipalities, it remains unclear whether they belong to a single landrace or to two distinct ones. However, it is known that farmers in that area have been cultivating *P. coccineus* genotypes since the early 1900s, and their produce is highly appreciated by local populations due to its organoleptic properties. 

For the identification of the collection sites of *P. coccineus* accessions, 32 smallholdings and/or family gardens were recognized in four different municipalities of the Aniene Valley (Figure S4) where *P. coccineus* genotypes are currently cultivated. Specifically, based on the names assigned by farmers, 16 accessions (populations) were collected for the Fagiolone landrace, of which 14 came from farms in the municipality of Vallepietra and 2 from the municipality of Subiaco, both of which are located in the province of Rome. Additionally, an equal number of accessions were collected for the Mandolone landrace, with 13 from farms in the municipality of Filettino and 3 from the municipality of Trevi nel Lazio, both of which are located in the province of Frosinone (Table S10). In this case, all the identified collection sites were georeferenced using the GPS, and the data were recorded in GIS. In addition, the farmers were interviewed to collect information regarding the cultivation time of *P. coccineus* genotypes, the sources of the seeds, their growth area, relative production, and traditional agriculture practices.

The seeds of the Fagiolone and Mandolone accessions analyzed in this study were collected during 2021 and 2022. Considering that *P. coccineus* is a predominantly outcrossing species, a sample of 100–300 seeds (depending on the cultivation area) was directly collected for each farm. Samples were taken from distinct plants, with each sample representing a unique population. After conducting quantitative morphological analyses, the seeds of each of the 32 *P. coccineus* accessions (populations) were divided into two lots and stored in the germplasm banks of ARSIAL and DIBAF. Ten of the seeds stored at DIBAF for each accession were used to obtain seedlings for DNA extraction, and we performed subsequent genetic characterization using SSR molecular markers. 

In the molecular analyses, seven accessions of *P. coccineus* with seed morphotypes, similar to the accessions collected in the Aniene Valley (uniformly white, large, and cuboid), were used as reference genotypes (Figure S2). Specifically, two of these accessions were related to the varieties Corona and Venere, which are registered in the national registry of vegetable varieties. The remaining five accessions belonged to landraces cultivated in Central Italy: Fagiolo di Grisciano (registered in the RVR) and Fagiolo della Nonna from the Lazio Region; Fagiolo di Campo di Giove from the Abruzzo region; Fagiolo di Pieve Torina from the Marche region; and Fagiolo di Spoleto from the Umbria region (Table S10). DNA was extracted from the leaves of five seedlings/plants for each of the seven accessions.

### 3.2. Seed Morphological Analysis

Considering that all 32 accessions of *P. coccineus* collected in the Aniene Valley are characterized by a very similar seed morphotype, using the descriptor list of the International Board for Plant Genetic Resources [29] (uniformly white, large, and cuboid), the following four quantitative traits related to seed size were determined for each accession: the weight of 100 seeds (g); the average height (mm), measured as the longest distance perpendicular to the length; the average length (mm), measured as the longest distance across the seed parallel to the hilum; the shape index, determined as the ratio between length and height (a ratio of 1 indicates a perfectly round seed). These measurements were performed on a representative sample of 20 seeds. 

### 3.3. DNA Extraction

DNA extraction from the *P. coccineus* seedlings was performed according to the work of Catarcione et al. [30], as were assessments of its integrity and concentration. For *P. coccineus*, a total of 355 DNA samples were extracted [(32 Aniene Valley accessions × 10 seedlings) + (7 control accessions × 5 seedlings)] and used for PCR amplifications with microsatellite markers.

### 3.4. Genetic Analysis Using SSR Molecular Markers

Since there are no SSR markers available that have been developed specifically for *P. coccineus*, we used nine [31] and three [32,33] SSR loci identified in *P. vulgaris*, covering coding and non-coding regions, respectively, to evaluate the inter- and intra-population diversity and genetic structure of the *P. coccineus* accessions collected in the Aniene Valley (Table S10). These SSR loci were selected based on their high values of PIC (Polymorphic Information Content), their chromosomal location (assigned to 10 out of the 11 linkage groups of *P. vulgaris*) and, in particular, their ability to discriminate among *P. coccineus* genotypes [2,34,35]. The characteristics of the 12 SSR markers are reported in Table S4.

### 3.5. Statistical Analysis

The mean and standard deviation values of 4 quantitative seed traits (weight of 100 seeds, height, length, and shape index) were calculated for each of the 16 accessions of Fagiolone and the 16 accessions of Mandolone and data were analyzed using ANOVA. Differences between the means of each landrace were compared using Tukey’s test with a significance level of *p* < 0.05. All statistical analyses were performed using JMP PRO 15 software.

The discriminant power of the SSR loci was assessed using the Polymorphic Information Content (PIC) index [36] employing the Power Marker 3.25 software [37]. We evaluated genetic diversity for each locus and landrace based on the following parameters: the number of alleles per locus (Na), the number of effective alleles (Ne), the number of rare (frequency < 0.05) and private alleles, major allele frequency (MAF), Shannon Index (I), the expected (He) and observed (Ho) heterozygosity, and the inbreeding coefficient (F). This work was performed using Power Marker 3.25 [37] and GenAlEx6 [38]. An analysis of molecular variance (AMOVA) was conducted to assess variance among and within populations using the GenAlEx6 software [38]. Variance components were statistically tested with non-parametric randomization tests using 999 permutations. 

The intra-population genetic variability was also analyzed through the inbreeding coefficient (Fis) [39] using FSTAT v2.9.3.2 [40]. Deviations from the Hardy–Weinberg (HE) equilibrium were assessed for each locus and population using the exact HW test employing the GENEPOP v. 3.4 software [41]. All analyses in GENEPOP were conducted with the following parameters: dememorization = 10000; number of batches = 100; number of iterations/batches = 1000. Significant positive Fis values indicate inbreeding (excess of homozygotes) or undetected null alleles, while significantly negative Fis values indicate an excess of heterozygosity and, consequently, a low level of inbreeding. Conversely, Fis values close to zero are expected under conditions of random mating.

The genetic distances for phylogenetic relationships among genotypes and accessions were estimated using the Nei coefficient [42]. Cluster analysis was performed using the Unweighted Pair-Group Method with Arithmetic Average (UPGMA) algorithm, and a dendrogram was generated using the Power Marker 3.25 software [37] and visualized using the MEGAX software [43]. 

The genetic relationships among *P. coccineus* accessions were also investigated through Principal Coordinates Analysis (PCoA) performed using GenAlEx6 software [37]. Finally, the fixation index Fst [39] was calculated to assess the level of genetic differentiation among different *P. coccineus* populations in the Aniene Valley using GenAlEx6 software [38].

To assess the genetic structure of *P. coccineus* landraces collected in the Aniene Valley, a Bayesian approach was employed using STRUCTURE software v. 2.3.4 [44], as described in [35]. The estimation of the most probable number of genetic groups in *P. coccineus* was performed using the method proposed by Evanno et al. [45], considering the highest ΔK statistic value and Ln Pr(K) values. Individuals with a membership probability Q > 0.75 were assigned to the corresponding genetic group.

Clustering, performed through UPGMA, PCoA, and STRUCTURE, allowed the classification of *P. coccineus* accessions into two groups related to the Fagiolone and Mandolone landraces. Genotypes considered to be of mixed origin (Q < 0.75) were excluded from subsequent analyses. The genetic diversity of each of the two groups identified was assessed by calculating the number of private alleles and the Na, Ne, He, and Ho using Power Marker 3.25 [37] and GenAlEx6 [38]. To test the significance of differences in genetic parameters between the two groups, the non-parametric Kruskal–Wallis test was performed using JMP PRO 15 software (©SAS Institute Inc., Cary, NC, USA). Additionally, molecular variance analysis (AMOVA) was performed, using GeneAlEx6 [38], to partition genetic variation between and within the two groups of genotypes. Variance components were statistically tested with non-parametric randomization tests using 999 permutations. 

## 4. Conclusions

This study reveals that, despite the high morphological uniformity observed in the seed traits of the 32 *Phaseolus coccineus* populations collected from the Aniene Valley, there is significant genetic diversity within these populations. This is an unexpected result, considering that *P. coccineus* is predominantly an outcrossing species. Molecular analyses categorized the population into two main genetic clusters: one related to the Mandolone accessions and the other to the Fagiolone accessions. Both were clearly distinct from the seven reference genotypes used in the analysis as a control.

Furthermore, the molecular data facilitated the accurate classification of accessions in terms of specific landraces, aiding in the management of genetically diverse resources within seemingly uniform materials. 

The analysis of the morphological and molecular traits of *Phaseolus coccineus* populations from the Aniene Valley revealed significant differences between the Mandolone and Fagiolone landraces. Mandolone accessions had higher seed weight and dimensions compared to Fagiolone accessions, although the L/H ratio did not differ significantly between the two. Molecular analysis using SSR markers demonstrated high genetic variability within these populations, with 53 alleles identified and high heterozygosity values expected. The genetic structure analysis revealed two distinct clusters corresponding to the Mandolone and Fagiolone landraces, with limited genetic differentiation between populations (Fst = 0.248). 

Despite the geographical proximity, the genetic distinctiveness of the Mandolone landrace suggests its potential for registration in the National Register of Biodiversity, managed by the Italian Ministry of Agriculture, Food and Forestry (Ministerial Decree no. 38,654 of 4 November 2019 and Ministerial Decree no. 13,073 of 17 April 2020). Such registration would not only facilitate the recognition of seeds and the identification of farmers cultivating this variety, but also make a significant contribution to germplasm conservation, helping to counteract the risk of the genetic erosion of native agricultural resources.

The findings of this study highlight the genetic richness of *P. coccineus* in the Aniene Valley and the necessity for targeted conservation efforts. The deviations observed from the Hardy–Weinberg equilibrium, potentially due to inbreeding, genetic drift, and farmer-driven selection, underline the need for strategies that maintain genetic diversity while supporting sustainable agricultural practices. The identification of two genetically distinct landraces provides a foundation for future breeding programs and the preservation of these genetic resources. Furthermore, the results support the development of strategies for both in situ/on-farm and ex situ conservation, including the establishment of a seed bank. These strategies are essential for both the preservation of the bean landraces of the Aniene Valley and for broader applications in managing genetic resources in legume crops. By enhancing seed multiplication practices and conserving genetic diversity, these approaches can be adapted to other regions, promoting the sustainability of local crop varieties in diverse agroecosystems and supporting their resilience in the face of environmental changes. 

## Figures and Tables

**Table 1:** Genetic diversity parameters found in the 32 *P. coccineus* populations collected in the Aniene Valley. ^a^ number of individuals; ^b^ number of alleles per locus; ^c^ number of private alleles; ^d^ Shannon Index; ^e^ observed heterozygosity; ^f^ expected heterozygosity; ^g^ inbreeding coefficient. An asterisk (*) indicates significance at *p* < 0.05.

Populations	Na ^a^	Ne ^b^	Npa ^c^	I ^d^	Ho ^e^	He ^f^	Fis ^g^
Vall_1	2.333	1.953	0	0.715	0.403	0.457	0.135 *
Vall_2	2.333	1.961	1	0.707	0.400	0.452	0.167 *
Vall_3	2.444	1.965	0	0.670	0.414	0.401	−0.036
Vall_4	2.333	1.920	0	0.649	0.400	0.395	0.04
Vall_5	2.444	1.939	0	0.716	0.413	0.453	0.127 *
Vall_6	2.889	2.148	0	0.820	0.491	0.484	−0.002
Vall_7	2.333	1.820	0	0.628	0.389	0.385	0.043
Vall_8	2.333	1.808	0	0.615	0.336	0.386	0.143 *
Vall_9	2.444	1.939	1	0.624	0.322	0.363	0.163 *
Vall_10	2.111	1.716	0	0.536	0.302	0.332	0.123 *
Vall_11	2.556	1.819	1	0.597	0.312	0.343	0.124 *
Vall_12	2.444	1.964	1	0.690	0.433	0.427	0.038
Vall_13	2.111	1.577	0	0.454	0.289	0.274	−0.002
Vall_14	2.333	1.854	1	0.604	0.378	0.363	0.013
Subi_1	2.444	2.133	0	0.783	0.437	0.503	0.154 *
Subi_2	2.444	2.042	0	0.745	0.511	0.476	−0.021
Fill_1	2.889	2.257	0	0.869	0.489	0.529	0.159 *
Fill_2	3.222	2.581	2	1.005	0.556	0.593	0.145 *
Fill_3	3	2.504	0	0.950	0.467	0.573	0.236 *
Fill_4	3	2.368	0	0.916	0.502	0.544	0.123 *
Fill_5	2.889	2.387	0	0.888	0.456	0.528	0.188 *
Fill_6	2.444	1.904	0	0.634	0.322	0.376	0.193 *
Fill_7	2.667	2.206	0	0.778	0.426	0.475	0.133 *
Fill_8	2.444	1.964	0	0.656	0.344	0.391	0.170 *
Fill_9	2.667	1.992	0	0.702	0.422	0.433	0.078
Fill_10	2.889	2.539	0	0.962	0.489	0.584	0.214 *
Fill_11	2.778	2.138	1	0.802	0.300	0.484	0.424 *
Fill_12	2.889	2.199	0	0.860	0.378	0.523	0.326 *
Fill_13	2.889	2.205	0	0.822	0.344	0.485	0.337 *
Trev_1	2.333	1.840	0	0.620	0.314	0.389	0.177 *
Trev_2	2	1.772	0	0.568	0.323	0.372	0.165 *
Trev_3	2.333	1.709	0	0.552	0.356	0.333	−0.016

**Table 2:** Analysis of molecular variance (AMOVA) among and within the 32 *P. coccineus* landraces collected in the Aniene Valley grouped based on their geographic origin. Statistical probability level P(*Φ*) with 999 permutations.

Source	df	SS	MS	Est. Var.	%	Fst	*Φ*-Statistic	P(*Φ*)
Population								
Among Pops	31	802.369	25.883	2.112	31%	0.248	0.307	<0.001
Within Pops	288	1370.400	4.758	4.758	69%			
Total	319	2172.769		6.871	100%			
Geographic origin								
Among Pops	3	466.889	155.630	2.233	29%	0.170	0.293	<0.001
Within Pops	316	1705.879	5.398	5.398	71%			
Total	319	2172.769		7.631	100%			

## Data Availability

The original contributions presented in this study are included in the article/Supplementary Material. Further inquiries can be directed to the corresponding author.

## Supplementary Materials

The following supporting information can be downloaded at https://www.mdpi.com/article/10.3390/plants13233320/s1, Figure S1. Distributions of the four quantitative seed traits among the 16 accessions of Fagiolone and the 16 accessions of Mandolone. Figure S2. Estimation of the optimal number of clusters for *P. coccineus* genotypes according to the Evanno method (2005). Above, the graphs display the DeltaK [mean(|L″(K)|)/sd(L(K))] for each value of K. Below, the statistics and results of the Evanno test for the STRUCTURE analysis. In yellow the optimal value of K is highlighted. Figure S3. Seeds from populations of Fagiolone and Mandolone landraces collected respectively from three farms in Vallepietra (RM) and two in Filettino (FR) compared with seeds from three accessions used as controls: the commercial variety “Corona” and the landraces collected in Grisciano in the province of Rieti in the Lazio Region and Spoleto in the province of Perugia in the Umbria region. Figure S4. Map of the Aniene Valley within the Lazio Region in which the four collection sites of *P. coccineus* accessions are indicated: Subiaco, Vallepietra, Filettino and Trevi nel Lazio. Table S1. Values of the four quantitative seed traits detected for the 16 accessions of Fagiolone and for the 16 accessions of Mandolone. L/H: length/height; SD: standard deviation; asterisks indicate significance levels as follows: * *p* < 0.05; ** *p* < 0.01; *** *p* < 0.001. Table S2. Genetic diversity parameters for the 9 SSRs used in the analyses of the *P. coccineus* collection in the Aniene Valley. Table S3. Rare and private alleles detected in the 32 *P. coccineus* accessions collected in the Aniene Valley, categorized as homozygous (Hom) or heterozygous (Het). The asterisk (*) denotes private alleles. In brackets are indicated the number of individuals of each accession in which the specific alleles were found. Table S4. Characteristics of the 12 SSR markers used in the analyses. Table S5. Pairwise Fst values of the *P. coccineus* populations. Table S6. Posterior membership coefficients following a STRUCTURE analysis of *P. coccineus* with K = 2. In yellow are indicated the seven genotypes of mixed origin. Table S7. Genetic diversity parameters of *P. coccineus* genotypes classified into two groups (Mesoamerican and Andean) according to the STRUCTURE analysis for K = 2. N: number of individuals for each group; Na: number of alleles per locus; Ne: number of effective alleles; Npa: number of private alleles; Ho: observed heterozygosity; He: expected heterozygosity. A significant level of *p* < 0.05 was used for the Kruskal–Wallis test. Table S8. List of private alleles detected in the Fagiolone and Mandolone populations. Table S9. Analysis of molecular variance (AMOVA) between and within the two groups of genotypes belonging to the Fagiolone and Mandolone landraces, identified through STRUCTURE analysis for K = 2. Statistical significance level P(Φ) with 999 permutations. Table S10. List of accessions of *P. coccineus* landraces collected in the Aniene Valley and of the landraces/varieties used as controls in the molecular analyses.
