# Supplementary material for: The Characterization of the Morphological and Molecular Traits of Phaseolus coccineus in the Aniene Valley: Insights into Genetic Diversity and Adaptation
Source: Plants (Basel). 2024 Nov 26;13(23):3320. doi: 10.3390/plants13233320 (PMC11644353; doi:10.3390/plants13233320)
Supplement: Supplementary file 1 [file plants-13-03320-s001.zip › Supplementary materials/Supplementary materials.pdf]

Supplementary materials

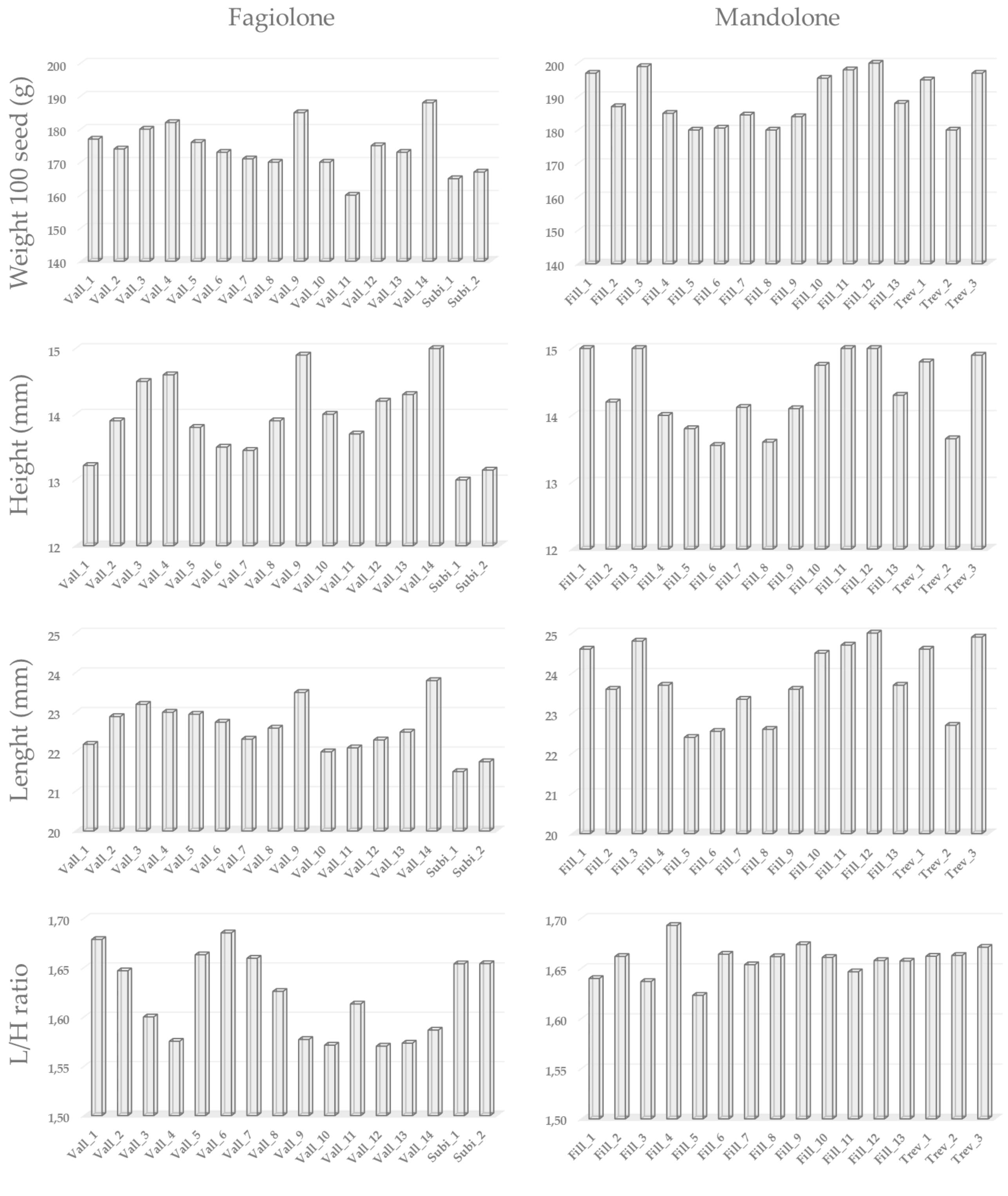

Figure S1. *Distributions of the four quantitative seed traits among the 16 accessions of Fagiolone and the 16 accessions of Mandolone*.

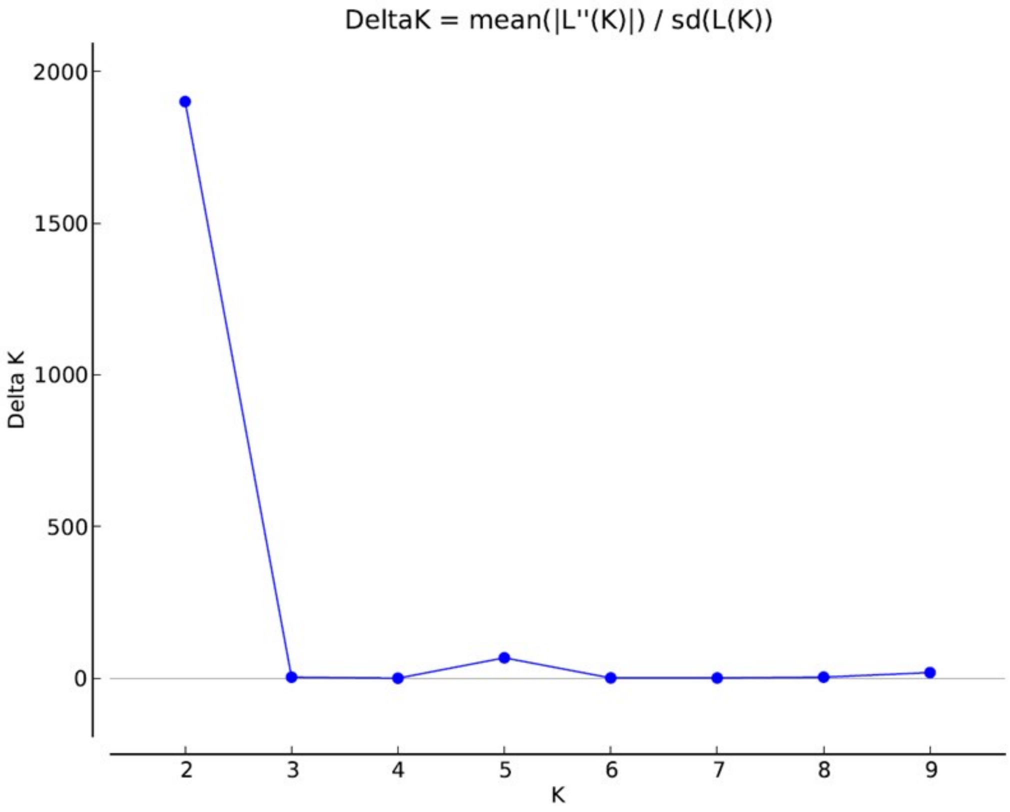

K	Reps	Mean LnP(K)	Stdev LnP(K)	Ln'(K)	|Ln''(K)|	Delta K
1	10	-5903.100000	0.210819	—	—	—
2	10	-4934.510000	0.384274	968.590000	730.500000	1900.986289
3	10	-4696.420000	30.322628	238.090000	103.450000	3.411644
4	10	-4561.780000	54.023468	134.640000	1.160000	0.021472
5	10	-4425.980000	1.144844	135.800000	77.030000	67.284305
6	10	-4367.210000	19.909096	58.770000	17.180000	0.862922
7	10	-4325.620000	34.869496	41.590000	26.000000	0.745637
8	10	-4258.030000	9.314391	67.590000	33.530000	3.599806
9	10	-4223.970000	12.483327	34.060000	231.640000	18.555951
10	10	-4421.550000	397.785996	-197.580000	—	—

Figure S2. *Estimation of the optimal number of clusters for P. coccineus genotypes according to the Evanno method (2005). Above, the graphs display the DeltaK [mean(|L''(K)|) / sd(L(K))] for each value of K. Below, the statistics and results of the Evanno test for the STRUCTURE analysis. In yellow the optimal value of K is highlighted.*

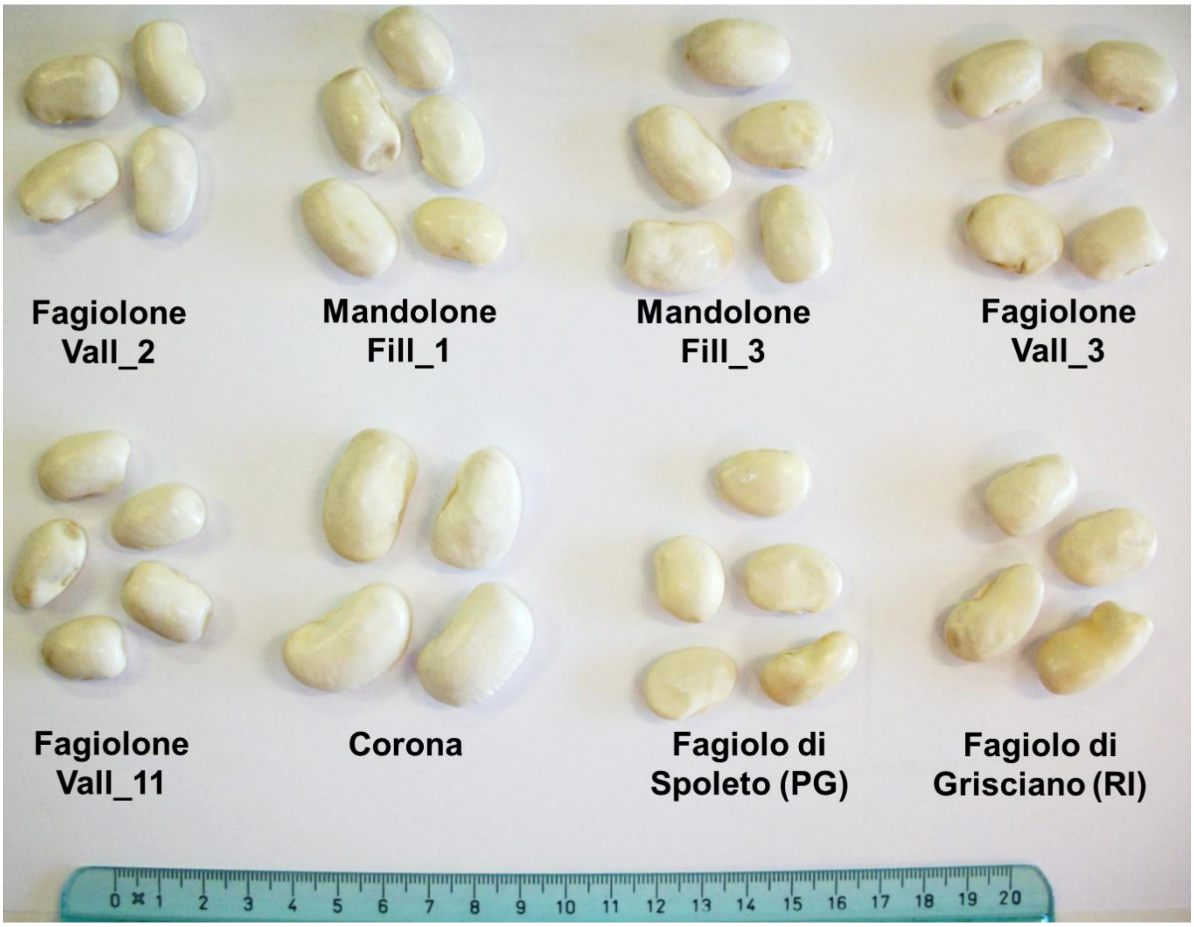

Figure S3. *Seeds from populations of Fagiolone and Mandolone landraces collected respectively from three farms in Vallepietra (RM) and two in Filettino (FR) compared with seeds from three accessions used as controls: the commercial variety "Corona" and the landraces collected in Grisciano in the province of Rieti in the Lazio region and Spoleto in the province of Perugia in the Umbria region.*

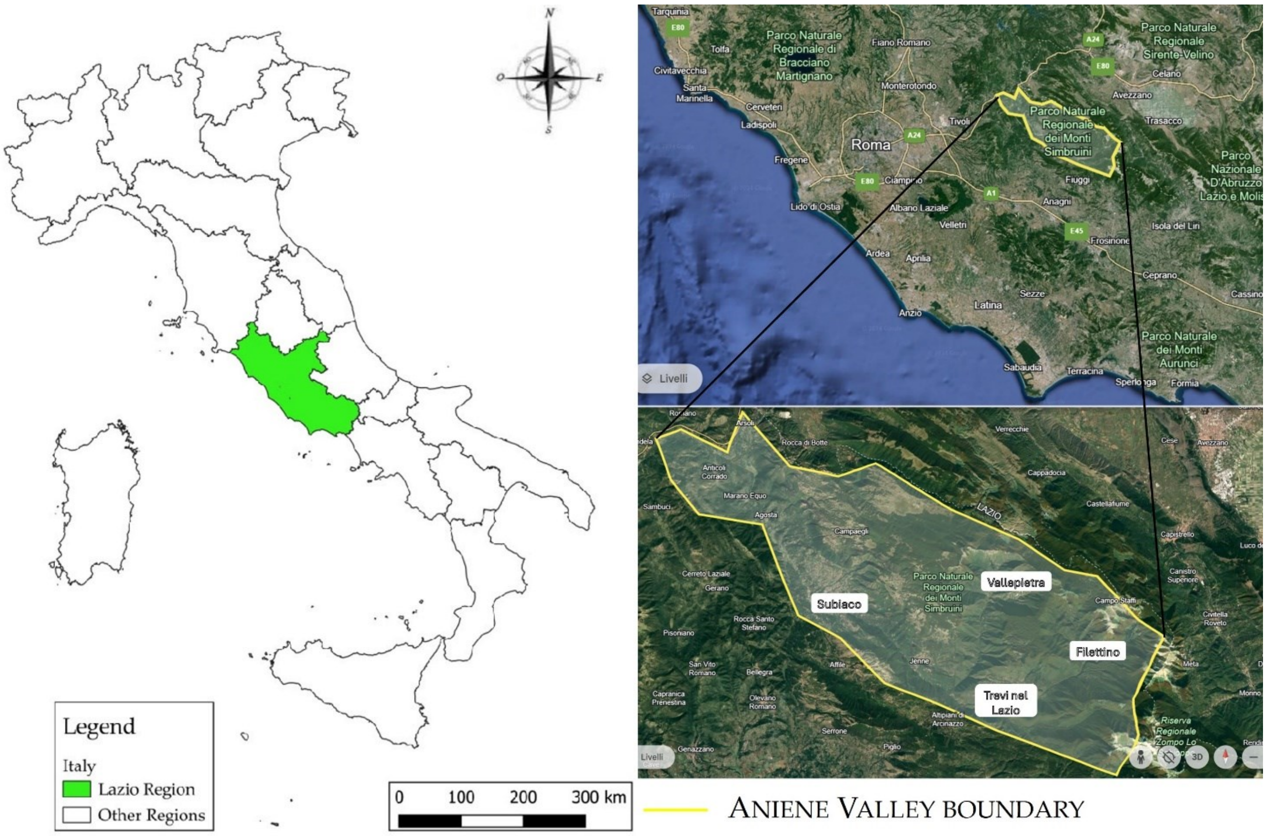

Figure S4. *Map of the Aniene Valley within the Lazio Region in which the four collection sites of P. coccineus accessions are indicated: Subiaco, Vallepietra, Filettino and Trevi nel Lazio.*

Table S1. *Values of the four quantitative seed traits detected for the 16 accessions of Fagiolone and for the 16 accessions of Mandolone. L/H: Length/Height; SD: standard deviation; asterisks indicate significance levels as follows: * p < 0.05; ** p < 0.01; *** p < 0.001.*

Landrace	Accession	Weight 100 seed (g)	Height (mm)	Length (mm)	L/H ratio
Fagiolone	Vall_1	177.00	13.22	22.19	1.68
Fagiolone	Vall_2	174.00	13.90	22.89	1.65
Fagiolone	Vall_3	180.00	14.50	23.20	1.60
Fagiolone	Vall_4	182.00	14.60	23.00	1.58
Fagiolone	Vall_5	176.00	13.80	22.95	1.66
Fagiolone	Vall_6	173.00	13.50	22.75	1.69
Fagiolone	Vall_7	171.00	13.45	22.32	1.66
Fagiolone	Vall_8	170.00	13.90	22.60	1.63
Fagiolone	Vall_9	185.00	14.90	23.50	1.58
Fagiolone	Vall_10	170.00	14.00	22.00	1.57
Fagiolone	Vall_11	160.00	13.70	22.10	1.61
Fagiolone	Vall_12	175.00	14.20	22.30	1.57
Fagiolone	Vall_13	173.00	14.30	22.50	1.57
Fagiolone	Vall_14	188.00	15.00	23.80	1.59
Fagiolone	Subi_1	165.00	13.00	21.50	1.65
Fagiolone	Subi_2	167.00	13.15	21.75	1.65
	Mean	174.13 ***	13.95 *	22.58 ***	1.62
	SD	7.30	0.61	0.63	0.04
Mandolone	Fill_1	197.00	15.00	24.60	1.64
Mandolone	Fill_2	187.00	14.20	23.60	1.66
Mandolone	Fill_3	199.00	15.15	24.80	1.64
Mandolone	Fill_4	185.00	14.00	23.70	1.69
Mandolone	Fill_5	180.00	13.80	22.40	1.62
Mandolone	Fill_6	180.60	13.55	22.55	1.66
Mandolone	Fill_7	184.50	14.12	23.35	1.65
Mandolone	Fill_8	180.00	13.60	22.60	1.66
Mandolone	Fill_9	184.00	14.10	23.60	1.67
Mandolone	Fill_10	195.50	14.75	24.50	1.66
Mandolone	Fill_11	198.00	15.00	24.70	1.65
Mandolone	Fill_12	201.00	15.20	25.20	1.66
Mandolone	Fill_13	188.00	14.30	23.70	1.66
Mandolone	Trev_1	195.00	14.80	24.60	1.66
Mandolone	Trev_2	180.00	13.65	22.70	1.66
Mandolone	Trev_3	197.00	14.90	24.90	1.67
	Mean	189.48 ***	14.38 *	23.84 ***	1.66
	SD	7.78	0.58	0.94	0.02

Table S2*. Genetic diversity parameters for the 9 SSRs used in the analyses of the P. coccineus collection in the Aniene Valley.*

Locus	Na[a]	Ne[b]	MAF[c]	PIC[d]	He[e]	Ho[f]	F[g]
X79722	4	2.197	0.294	0.758	0.788	0.528	0.330
U18349	2	1.548	0.631	0.446	0.516	0.278	0.461
X04660	5	2.705	0.522	0.614	0.656	0.919	-0.401
M75856	7	2.723	0.231	0.815	0.836	0.666	0.204
X60000	6	2.317	0.425	0.708	0.741	0.219	0.705
AF483902	6	2.158	0.475	0.672	0.706	0.275	0.610
X04001	6	1.692	0.656	0.507	0.536	0.163	0.697
X74919	7	1.944	0.609	0.578	0.600	0.209	0.651
X80051	10	3.925	0.272	0.867	0.876	0.397	0.547
Mean	5.89	2.357	0.457	0.663	0.695	0.406	0.423
Total	53.0						

[a] Number of alleles per locus; [b] Number of effective alleles; [c] Major Allele Frequency; [d] Polymorphic Information Content; [e] Expected heterozygosity; [f] Observed heterozygosity; [g] Inbreeding coefficient.

***Table S3**. Rare and private alleles detected in the 32 P. coccineus accessions collected in the Aniene Valley, categorized as homozygous (Hom) or heterozygous (Het). The asterisk (*) denotes private alleles. In brackets are indicated the number of individuals of each accession in which the specific alleles were found.*

LOCUS	Allele	Frequency	Accession name	ARSIAL Code	Landrace	Status
X04660	204	0.008	Vall_11 (3)	VE-0671	Fagiolone	Het
			Vall_6 (2)	VE-0696	Fagiolone	Het
M75856	138	0.027	Subi_2 (1)	VE-0710	Fagiolone	Het
			Fill_5 (2)	VE-0684	Mandolone	Hom/Het
			Fill_2 (5)	VE-0674	Mandolone	Hom/Het
			Fill_4 (3)	VE-0679	Mandolone	Het
			Fill_1 (1)	VE-0672	Mandolone	Het
			Fill_13 (1)	VE-0776	Mandolone	Hom
			Fill_11 (1)	VE-0774	Mandolone	Het
	134*	0.002	Vall_9	VE-0699	Fagiolone	Het
	142*	0.002	Vall_14	VE-0703	Fagiolone	Het
X60000	125	0.017	Vall_3 (2)	VE-0693	Fagiolone	Het
			Vall_4 (2)	VE-0694	Fagiolone	Het
			Vall_7 (1)	VE-0697	Fagiolone	Het
			Vall_11 (2)	VE-0671	Fagiolone	Het
			Vall_14 (2)	VE-0703	Fagiolone	Het
			Vall_8 (1)	VE-0698	Fagiolone	Het
			Vall_13 (1)	VE-0702	Fagiolone	Het
	127*	0.002	Vall_12	VE-0701	Fagiolone	Het
	137*	0.006	Fill_2 (2)	VE-0674	Mandolone	Hom
AF483902	152*	0.011	Fill_2 (6)	VE-0674	Mandolone	Hom/Het
	158	0.016	Trev_1 (2)	VE-0687	Mandolone	Hom/Het
			Trev_3 (2)	VE-0779	Mandolone	Hom/Het
			Trev_2 (2)	VE-0688	Mandolone	Hom
	172*	0.002	Vall_2	VE-0626	Fagiolone	Hom
X04001	152	0.020	Vall_3 (1)	VE-0693	Fagiolone	Het
			Vall_5 (2)	VE-0695	Fagiolone	Het
			Vall_1 (3)	VE-0575	Fagiolone	Het
			Subi_2 (4)	VE-0710	Fagiolone	Het
			Subi_1 (2)	VE-0709	Fagiolone	Het
			Vall_6 (1)	VE-0696	Fagiolone	Het
	154	0.030	Vall_6 (1)	VE-0696	Fagiolone	Het
			Fill_2 (4)	VE-0674	Mandolone	Het
			Fill_3 (3)	VE-0676	Mandolone	Het
			Fill_10 (4)	VE-0681	Mandolone	Het
			Fill_4 (3)	VE-0679	Mandolone	Het
			Fill_1 (4)	VE-0672	Mandolone	Het
	158	0.020	Vall_5 (1)	VE-0695	Fagiolone	Hom
			Vall_12 (3)	VE-0701	Fagiolone	Hom/Het
			Vall_2 (4)	VE-0626	Fagiolone	Hom/Het
	164*	0.002	Vall_11	VE-0671	Fagiolone	Het
X74919	147*	0.002	Fill_11	VE-0774	Mandolone	Het
	155	0.044	Vall_5 (5)	VE-0695	Fagiolone	Hom/Het

			Vall_1 (5)	VE-0575	Fagiolone	Hom/Het
			Subi_2 (3)	VE-0710	Fagiolone	Het
			Subi_1 (6)	VE-0709	Fagiolone	Hom/Het
			Vall_6 (2)	VE-0696	Fagiolone	Het
	159	0.025	Fill_3 (1)	VE-0676	Mandolone	Hom
			Fill_10 (2)	VE-0681	Mandolone	Hom
			Trev_3 (1)	VE-0779	Mandolone	Hom
			Trev_1 (4)	VE-0687	Mandolone	Hom/Het
			Fill_12 (1)	VE-0775	Mandolone	Hom
	161	0.005	Vall_8 (1)	VE-0698	Fagiolone	Het
			Vall_9 (2)	VE-0699	Fagiolone	Het
X80051	214	0.042	Fill_9 (1)	VE-0680	Mandolone	Hom
			Fill_7 (1)	VE-0776	Mandolone	Hom
			Fill_2 (4)	VE-0674	Mandolone	Hom/Het
			Fill_4 (2)	VE-0679	Mandolone	Hom/Het
			Fill_1 (4)	VE-0672	Mandolone	Hom/Het
			Fill_12 (4)	VE-0775	Mandolone	Hom/Het
			Fill_13 (4)	VE-0776	Mandolone	Hom/Het
	216	0.027	Vall_11 (1)	VE-0671	Fagiolone	Het
			Vall_14 (2)	VE-0703	Fagiolone	Het
			Vall_13 (1)	VE-0702	Fagiolone	Het
			Fill_8 (1)	VE-0777	Mandolone	Het
			Fill_6 (3)	VE-0775	Mandolone	Hom/Het
			Fill_3 (3)	VE-0676	Mandolone	Hom/Het
			Fill_10 (3)	VE-0681	Mandolone	Hom/Het
	218	0.023	Vall_9 (3)	VE-0699	Fagiolone	Hom/Het
			Fill_9 (1)	VE-0680	Mandolone	Het
			Fill_7 (3)	VE-0776	Mandolone	Het
			Fill_5 (4)	VE-0684	Mandolone	Hom/Het
	228	0.011	Fill_9 (3)	VE-0680	Mandolone	Hom/Het
			Fill_7 (2)	VE-0776	Mandolone	Hom/Het
	230	0.008	Fill_2 (1)	VE-0674	Mandolone	Het
			Trev_3 (3)	VE-0779	Mandolone	Het
			Trev_1 (1)	VE-0687	Mandolone	Het
	224	0.038	Vall_3 (3)	VE-0693	Fagiolone	Hom/Het
			Vall_4 (3)	VE-0694	Fagiolone	Hom/Het
			Vall_7 (3)	VE-0697	Fagiolone	Hom/Het
			Vall_8 (5)	VE-0698	Fagiolone	Hom/Het
			Vall_9 (2)	VE-0699	Fagiolone	Hom/Het
			Trev_1 (1)	VE-0687	Mandolone	Het
			Fill_13 (1)	VE-0776	Mandolone	Het

Table S4. *Characteristics of the 12 SSR markers used in the analyses.*

SSR code	Genebank entry	Description	LG	Forward primer	Reverse primer	Motif	Annealing T (°C)	Dye color	References
PVcct001	X79722	Acyl-ACP,plsB mRNA	B02	CCAACCACATTCTTCCCTACGTC	GCGAGGCAGTTATCTTTAGGAGTG	$(CCT)_7$	58	FAM	Yu et al (2000)
BMd-20	X74919	Endochitinase	B05	CCGTTGCCTGTATTTCCCCAT	CTGGTGAAGTCATCTGGAGTGGTC	$(AT)_5$	49	JOE	Yu et al (2000)
PV-at007	X80051	NADP-dependent malic enzyme	B09	AGTTAAATTATACGAGGTTAGCCTAAATC	CATTCCCTTCACACATTCACCG	$(AT)_{12}$	49	TAMRA	Yu et al (2000)
PV-ag001	M75856	Pathogenesis-related protein 3	B11	CAATCCTCTCTCTCTCATTTCCAATC	GACCTTGAAGTCGGTGTCGTTT	$(GA)_{11}$	49	FAM	Yu et al (2000)
BMd-44	AZ301573	*P. vulgaris* genomic clone pBng 125	B08	GGCAGCTTACTAACCCGAAA	TTCCTTCCCCTTTCTTCTCC	$(AG)_5$	57	JOE	Blair et al (2003)
BMd-1	X96999	Pathogenesis-related protein 10	B03	AGTCGCCATAGTTGAAATTTAGGTG	CTTATTAAAACGTGAGCATATGTATCATTC	$(AT)_9$	58	TAMRA	Yu et al (2000)
BMd-41	AZ301561	*P. vulgaris* genomic clone pBng 91	B10	CAGTAAATATTGGCGTGGATGA	TGAAAGTGCAGAGTGTGTGGA	$(ATT)_9$	57	FAM	Blair et al (2003)
PV-at003	X60000	Small subunit of ribulose bisphosphate carboxylase/oxygenase	B04	ACCTAGAGCCTAATCCTTCTGCGT	GAATGTGAATATCAGAAAGCAAATGG	$(AT)_4(T)_2$	49	JOE	Yu et al (2000)
PV-ag004	X04660	Pseudogene Pdlec1 for phytohemagglutinin	B04	TTGATGACGTGGATGCATTGC	AAAGGGCTAGGGAGAGTAAGTTGG	$(AG)_8$	57	TAMRA	Yu et al (2000)
PV-ag003	X04001	Glutamine synthetase	B01	TCACGTACGAGTTGAATCTCAGGAT	GGTGTCGGAGAGGTTAAGGTTG	$(AG)_8$	49	FAM	Yu et al (2000)
U18349	U18349	Phaseolin G-box binding protein	B02	CTGAAGCCCGAATCTTGCGA	CGCGAGAGGTGAACGAAAGC	$(GGC)_5$	57	JOE	Yu et al (2000)
BM210	AF483902	*P. vulgaris* genomic clone D922	B07	CCCTCATCCTCCATTCTTATCG	ACCACTGCAATCCTCATCTTTG	$(CT)_{15}$	52	TAMRA	Gaitan-Solis et al (2002)

Table S7. *Genetic diversity parameters of P. coccineus genotypes classified into two groups (Mesoamerican and Andean) according to the STRUCTURE analysis for K=2. N: number of individuals for each group; Na: number of alleles per locus; Ne: number of effective alleles; Npa: number of private alleles; Ho: observed heterozygosity; He: expected heterozygosity. A significant level of p<0.05 was used for the Kruskal-Wallis test.*

**Cluster**	**N**	**Na**	**Ne**	**Npa**	**Ho**	**He**
**Fagiolone**	157	4.444	2.093	13	0.400	0.460
**Mandolone**	156	4.444	2.627	13	0.412	0.577
***p* value**<br>**Test Kruskal-Wallis**					0.822	0.289

Table S8. *List of private alleles detected in the Fagiolone and Mandolone populations.*

Pop	Locus	Allele	Freq
Fagiolone	X79722	138	0.411
	X04660	204	0.013
	X04660	208	0.162
	M75856	134	0.003
	M75856	142	0.003
	X60000	125	0.035
	X60000	127	0.003
	AF483902	172	0.006
	X04001	152	0.041
	X04001	158	0.041
	X04001	164	0.003
	X74919	155	0.089
	X74919	161	0.010
Mandolone	X79722	140	0.407
	X79722	146	0.119
	X60000	137	0.013
	AF483902	152	0.022
	AF483902	158	0.032
	X04001	154	0.058
	X04001	166	0.417
	X74919	147	0.003
	X74919	157	0.109
	X74919	159	0.051
	X80051	214	0.087
	X80051	228	0.022
	X80051	230	0.016

Table S9. *Analysis of Molecular Variance (AMOVA) between and within the two groups of genotypes belonging to the Fagiolone and Mandolone landraces, identified through STRUCTURE analysis for K=2. Statistical significance level P(Φ) with 999 permutations.*

Source	df	SS	MS	Est. Var.	%	Fst	*Φ*-Statistic	P(*Φ*)
**Among Groups**	1	356.733	356.733	2.243	28%	0.109	0.28	<0.001
**Within Groups**	311	1777.765	5.716	5.716	72%			
**Total**	312	2134.498		7.959	100%			

Table S10. *List of accessions of P. coccineus landraces collected in the Aniene Valley and of the landraces/varieties used as controls in the molecular analyses.*

No	Landrace	ARSIAL code	DIBAF code	Municipality
1	Fagiolone	VE-0575	Vall_1	Vallepietra (RM)
2	Fagiolone	VE-0626	Vall_2	Vallepietra (RM)
3	Fagiolone	VE-0693	Vall_3	Vallepietra (RM)
4	Fagiolone	VE-0694	Vall_4	Vallepietra (RM)
5	Fagiolone	VE-0695	Vall_5	Vallepietra (RM)
6	Fagiolone	VE-0696	Vall_6	Vallepietra (RM)
7	Fagiolone	VE-0697	Vall_7	Vallepietra (RM)
8	Fagiolone	VE-0698	Vall_8	Vallepietra (RM)
9	Fagiolone	VE-0699	Vall_9	Vallepietra (RM)
10	Fagiolone	VE-0670	Vall_10	Vallepietra (RM)
11	Fagiolone	VE-0671	Vall_11	Vallepietra (RM)
12	Fagiolone	VE-0701	Vall_12	Vallepietra (RM)
13	Fagiolone	VE-0702	Vall_13	Vallepietra (RM)
14	Fagiolone	VE-0703	Vall_14	Vallepietra (RM)
15	Fagiolone	VE-0709	Subi_1	Subiaco (RM)
16	Fagiolone	VE-0710	Subi_2	Subiaco (RM)
17	Mandolone	VE-0672	Fill_1	Filettino (FR)
18	Mandolone	VE-0674	Fill_2	Filettino (FR)
19	Mandolone	VE-0676	Fill_3	Filettino (FR)
20	Mandolone	VE-0679	Fill_4	Filettino (FR)
21	Mandolone	VE-0684	Fill_5	Filettino (FR)
22	Mandolone	VE-0775	Fill_6	Filettino (FR)
23	Mandolone	VE-0776	Fill_7	Filettino (FR)
24	Mandolone	VE-0777	Fill_8	Filettino (FR)
25	Mandolone	VE-0680	Fill_9	Filettino (FR)
26	Mandolone	VE-0681	Fill_10	Filettino (FR)
27	Mandolone	VE-0774	Fill_11	Filettino (FR)
28	Mandolone	VE-0775	Fill_12	Filettino (FR)
29	Mandolone	VE-0776	Fill_13	Filettino (FR)
30	Mandolone	VE-0687	Trev_1	Trevi nel Lazio (FR)
31	Mandolone	VE-0688	Trev_2	Trevi nel Lazio (FR)
32	Mandolone	VE-0779	Trev_3	Trevi nel Lazio (FR)
33	Fagiolo di Grisciano	4392	Grisciano_RI	Grisciano (RI)
34	Fagiolo della Nonna	VE-0778	SBiagioS_FR	San Biagio Saracinisco (FR)
35	Corona	Corona	Corona	Colfiorito (PG)
36	Venere	Venere	Venere	Colfiorito (PG)
37	Fagiolo di Campo di Giove	PHA_8285	Cgiove_AQ	Campo di Giove (AQ)
38	Fagiolo di Pieve Torina	Acc_257	PieveT_MC	Pieve Torina (MC)
39	Fagiolo di Spoleto	3615	Spoleto_PG	Spoleto (PG)
